# Teleassistance Telerehabilitation Services for urgent mental health needs of people with Intellectual and Developmental disabilities

**DOI:** 10.1192/j.eurpsy.2024.55

**Published:** 2024-08-27

**Authors:** M. O. Bertelli

**Affiliations:** CREA (Research and Clinical Centre), San Sebastiano Foundation, Misericordia di Firenze, Florence, Italy

## Abstract

**Abstract:**

Persons with intellectual disability (PwID) and/or and autism spectrum disorder with high support needs (ASD-HSN) have resulted to be among the most vulnerable populations to COVID-19 and distress factors associated to the measures for containing its spread. Many health, rehabilitation, and assistance needs were managed through the use of telemedicine, specifically teleassistance (TA) and telerehabilitation (TR), with regard to the prevention and treatment of the epidemic illness as well as the continuity of care required for the condition of developmental disability and co-occurring physical or mental disorders. TA and TR can function either directly or indirectly with the PwID/ASD; in the latter case, a family member, a regular caregiver, or a technician provides local mediation. This paper examines the most common TA and TR activities, along with their requirements, applications, and goals. All of these activities should be in line with the overarching goal of each customized therapy and rehabilitation plan, which is to enhance and support the quality of life for people with intellectual and developmental disabilities.

Studies on TA and TR efficacy for PwID/ASD are limited, especially concerning adulthood. The scant research that is currently available demonstrates efficacy in maintaining or marginally enhancing cognitive, adaptive, and vocational skills. In addition to managing both routine and unusual activities as well as critical episodes, family members and other caregivers reported feeling more empowered about their educational and interpersonal abilities with the PwID/ASD. The primary benefits over traditional in-person services have been found to be greater accessibility and availability as well as a reduction in both physical and psychological distance. The primary limits include the absence of all elements of the therapeutic alliance pertaining to face-to-face communication, possible poor ability to use technology, accessibility of the technology itself, concerns regarding privacy, and variables that divert attention connected to the household setting.

Even while telemedicine has proven to be feasible and beneficial thus far, it is doubtful that traditional techniques will be able to be replaced, at least not anytime soon. Telemedicine could, nonetheless, serve as a useful addition, integration, or short-term substitute. Future studies should provide light on the indications, contextual deployment, efficacy evaluation, and operational stability over time of certain TA and TR activities in addition to the use of artificial intelligence, machine learning, and interactive avatars.

**Disclosure of Interest:**

None Declared

